# Correction: Seo et al. A Timeline of Biosynthetic Gene Cluster Discovery in *Aspergillus fumigatus*: From Characterization to Future Perspectives. *J. Fungi* 2024, *10*, 266

**DOI:** 10.3390/jof10090657

**Published:** 2024-09-18

**Authors:** Hye-Won Seo, Natalia S. Wassano, Mira Syahfriena Amir Rawa, Grant R. Nickles, André Damasio, Nancy P. Keller

**Affiliations:** 1 Department of Medical Microbiology and Immunology, University of Wisconsin, Madison, WI 53706, USA; hseo45@wisc.edu (H.-W.S.); wassano@wisc.edu (N.S.W.); bintiamirraw@wisc.edu (M.S.A.R.); gnickles@wisc.edu (G.R.N.); 2 Department of Biochemistry and Tissue Biology, Institute of Biology, University of Campinas (UNICAMP), São Paulo 13083-970, Brazil; adamasio@unicamp.br; 3 Department of Plant Pathology, University of Wisconsin, Madison, WI 53706, USA

## Error in Figure

In the original publication [1], there was a mistake in Figure 6, “Novel BGCs identified in this study”, as published. In the original publication, we reported the identification of 20 novel biosynthetic gene clusters (BGCs) in *Aspergillus fumigatus* based on automated analyses using BiG-SCAPE, antiSMASH, and cBlaster. A subsequent detailed manual review revealed that only four of the initially identified clusters are novel. The corrected Figure 6 appears below.

## Text Correction

We have made edits in all sections referencing Figure 6 and the novel GCFs. A correction has been made in the following locations.

The final sentences in the abstract referencing our analysis of 264 genomes of *A. fumigatus* have been updated to reflect that we located four, not twenty, novel BGCs.

*Corrected paragraph:*

“**Abstract:** In 1999, the first biosynthetic gene cluster (BGC), synthesizing the virulence factor DHN melanin, was characterized in *Aspergillus fumigatus*. Since then, 19 additional BGCs have been linked to specific secondary metabolites (SMs) in this species. Here, we provide a comprehensive timeline of *A. fumigatus* BGC discovery and find that initial advances centered around the commonly expressed SMs where chemical structure informed rationale identification of the producing BGC (e.g., gliotoxin, fumigaclavine, fumitremorgin, pseurotin A, helvolic acid, fumiquinazoline). Further advances followed the transcriptional profiling of a Δl*aeA* mutant, which aided in the identification of endocrocin, fumagillin, hexadehydroastechrome, trypacidin, and fumisoquin BGCs. These SMs and their precursors are the commonly produced metabolites in most *A. fumigatus* studies. Characterization of other BGC/SM pairs required additional efforts, such as induction treatments, including co-culture with bacteria (fumicycline/neosartoricin, fumigermin) or growth under copper starvation (fumivaline, fumicicolin). Finally, four BGC/SM pairs were discovered via overexpression technologies, including the use of heterologous hosts (fumicycline/neosartoricin, fumihopaside, sphingofungin, and sartorypyrone). Initial analysis of the two most studied *A. fumigatus* isolates, Af293 and A1160, suggested that both harbored ca. 34–36 BGCs. An examination of 264 available genomes of *A. fumigatus* located only four additional new BGCs, suggesting the secondary metabolome across *A. fumigatus* isolates is remarkably conserved. Based on our analysis, around 20 of the genetically characterized BGCs within the *A. fumigatus* species complex still lack a known chemical product. Such BGCs remain the final hurdle in fully understanding the secondary metabolism in this important species.”

The method section titled “*Grouping the A. fumigatus BGC Predictions into Gene Cluster Families*” was adjusted to include the new manual inspection that was conducted on all GCFs.

*Corrected paragraph:*

*2.5. Grouping the A. fumigatus BGC Predictions into Gene Cluster Families*

Homologous BGCs are thought to produce identical or closely related SMs and are referred to as gene cluster families (GCFs) [17]. To determine which of our detected BGCs were members of shared GCFs, all antiSMASH predictions were run through BiG-SCAPE (e.g., Biosynthetic Gene Similarity Clustering and Prospecting Engine; v1.1.5) [18]. A total of seven BiG-SCAPE cutoff values between 0.1 and 0.7 by increments of 0.1 were tested. Values greater than 0.5 were found to be too relaxed, leading to the major merging of large GCFs, which were separated at lower cutoffs. In the end, an optimal cutoff value of 0.3 (which is also the default value) was chosen for generating initial GCF classifications. A network visualization was created with Cytoscape (v3.9.1) [19] for each natural product class and can be seen in Supplementary Figure S2. Each GCF generated by BiG-SCAPE was manually inspected to confirm that its genetic components did not resemble any known BGCs. This re-evaluation uncovered that some originally “rare” GCFs were products of isolate-to-isolate discrepancies in orthologous BGC boundaries identified by antiSMASH. Furthermore, it was observed that BGCs exhibiting features of various biosynthetic types often corresponded to GCFs spanning multiple biosynthetic classes. A plot visualizing the four GCFs that we believe are novel to this study can be found in Figure 6.

In the second and third paragraphs of Section 3.3, “20” should be changed to “22”.

The paragraph in the results section referencing Figure 6 has been adjusted to indicate that we found four novel BGCs in this study.

*Corrected paragraph:*

“Beyond the BGCs described in Lind et al. (2017) [117], we only found four BGCs that we confidently felt could be new clusters. Figure 6 visually depicts four GCFs considered novel in this research”.

The final section in the discussion section has been altered to place a larger emphasis on the BGCs with known products and those with known genetics but unknown chemical products.

*Corrected paragraph:*

“Our analysis reveals a notably conserved secondary metabolome across *A. fumigatus* isolates. All but four of the BGCs that we identified either correlate with known chemical products (*n* = 22) or have been previously genetically characterized but remain unlinked to any natural product production (*n* = 18). Fully characterizing all BGCs with yet unidentified natural products will likely necessitate the use of overexpression technologies, whether endogenous or heterologous. However, the potential for discovering novel chemistries lies within the extensive array of putative BGCs that have yet to be chemically defined (Figure 5)”.

Removed References 119, 127 and 128 from the original manuscript. With this correction, the order of some references has been adjusted accordingly.

The authors state that the scientific conclusions are unaffected. This correction was approved by the Academic Editor. The original publication has also been updated.

## Figures and Tables

**Figure 6:**
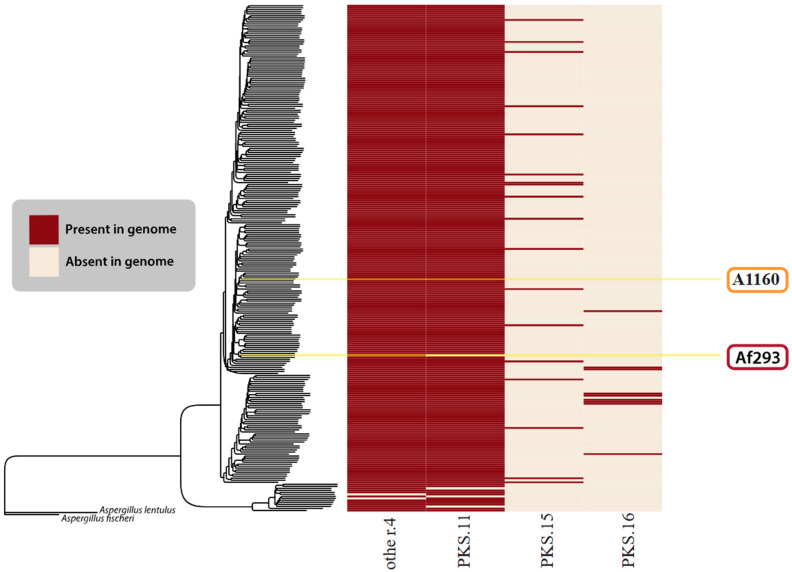
Novel BGCs identified in this study. The species tree is identical to that in Figure 5. The heatmap shows the presence (red) or absence (cream) of all novel/unknown GCFs found in the *A. fumigatus* isolates.
