# Supplementary material for: A Timeline of Biosynthetic Gene Cluster Discovery in Aspergillus fumigatus: From Characterization to Future Perspectives
Source: J Fungi (Basel). 2024 Apr 2;10(4):266. doi: 10.3390/jof10040266 (PMC11051388; doi:10.3390/jof10040266)
Supplement: Supplementary file 1 [file jof-10-00266-s001.zip › SupFigures_JoF.pdf]

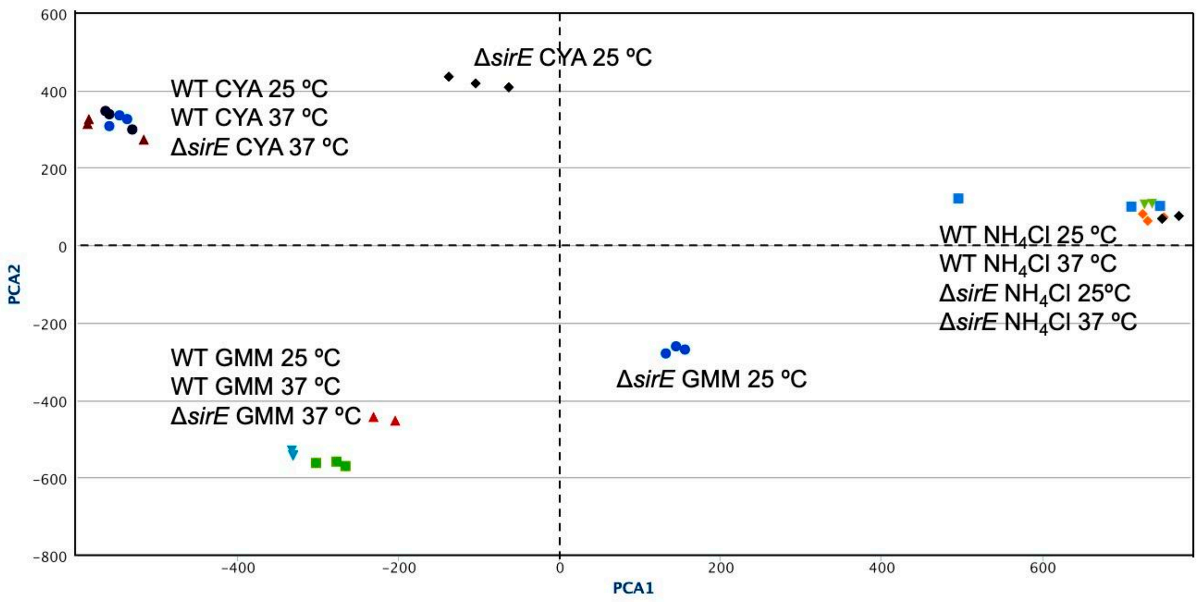


**Supplemental Figure S1. Distributions of SM features in *A. fumigatus* WT and Δ*sirE* strains based on the principal component analysis.** Five main clusters were observed by which the *Δsir E* strain grown at 25 °C in both CYA and GMM were clustered individually and separately from the rest of the growth conditions.

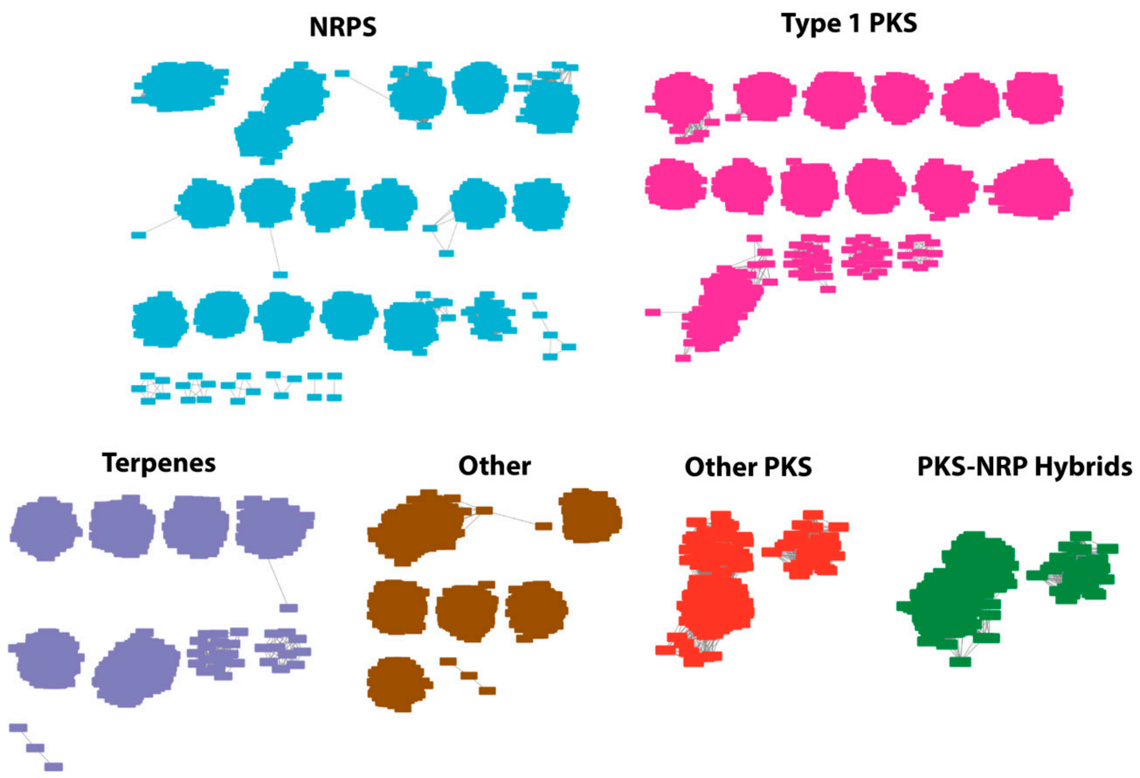


**Supplemental Figure S2. Network visualization of the secondary metabolome in the *Asper gillus fumigatus* species complex.** The classes are defined by the presence of core natural pro duct synthases/synthetases within the detected biosynthetic gene cluster. Each class of secon dary metabolites is colored and labeled accordingly.
